# Packing a Punch: Fight Bite Induced Septic Joint

**DOI:** 10.7759/cureus.30765

**Published:** 2022-10-27

**Authors:** Mohammad Anzal Rehman, Mohammad Issa Naser, Omnia B Ali, Patrick Ukwade

**Affiliations:** 1 Emergency Department, Mediclinic City Hospital, Dubai, ARE; 2 Emergency Department, Zayed Military Hospital, Abu Dhabi, ARE; 3 Emergency Department, Gulf Medical University, Ajman, ARE; 4 Emergency Department, Mayo Clinic - Sheikh Shakbout Medical City, Abu Dhabi, ARE

**Keywords:** antibiotic prophylaxis, wound care, flexor tenosynovitis, septic arthritis, fight bite

## Abstract

‘Fight bites’ constitute a considerable number of accidental human bite injuries. Where the mechanism involves a closed fist contacting another person’s teeth, the subsequent injury tends to involve the metacarpophalangeal joint region. These injuries are unique for their seemingly benign appearance on initial presentation. Their presence can easily be missed if the treating physician does not seek investigative history and a high index of suspicion. If improperly managed, fight bites may be associated with the introduction of bacteria that may invade deeper tissues, causing potential debilitation from progressive infection.

Our case discusses a 33-year-old female who presented three weeks after an altercation where a fight bite occurred but was not treated with antibiotics on discharge. Her clinical presentation matched a flexor sheath infection, which was revealed after investigation to be a consequence of a septic metacarpophalangeal joint that had also progressed to involve the underlying bones. The case outlines the dangers of improper assessment and management of fight bite injuries and reviews best practices surrounding the recognition, assessment, and treatment of these patients in the Emergency Department.

## Introduction

‘Fight bites’ are described as human bite wounds sustained during altercations where the victim injures an extremity; usually, a hand closed into a fist through impact against another person’s teeth [1]. These injuries must be examined thoroughly, and an emphasis should be made on avoiding the dissemination of bacterial infection from these wounds. Improper management of wound neglect and inadequate antibiotic coverage may lead to severe infections that can infiltrate deeper tissues and bone [2].

In this case, we discuss an adult female patient who presented to our emergency department (ED) 3 weeks after an initial fight bite injury, not treated with antibiotic therapy on discharge, with symptoms of flexed, swollen ring finger with pain on extension, found on X-ray to demonstrate bony effacement and periosteal elevation in the underlying MCP. We highlight key elements in wound care for such patients and elaborate on risk factors associated with the outcomes seen in this case.

## Case presentation

A 33-year-old female presented to a tertiary care hospital ED with complaints of swelling to the ring finger of her right hand for the past week. As described by the patient, she was involved in a physical altercation three weeks prior while confined in a jail cell with another inmate.

During the fight, the patient punched the other inmate using her right hand, impacting the person’s teeth and metallic dental braces. Consequently, the patient had pain and a small laceration at the dorsal aspect of the right-hand ring finger at the level of the Metacarpophalangeal (MCP) joint. The hand and its wound were inspected at a local clinic. Only irrigation was done for the seemingly minor laceration (less than 1 cm), and the patient was discharged back to her cell without any antibiotic coverage.

Approximately one week after the injury, the patient revisited a clinic for complaints of increasing pain and swelling over the dorsal aspect of the right-hand ring finger, located around the region of her fight bite wound. This time, the patient was discharged with oral antibiotics (Amoxicillin-Clavulanate).

One day before arrival in ED, the patient reported progressively worsening pain and swelling over the right ring finger, now associated with erythema and reduced range of movements over the joint. An X-ray of the right hand was unremarkable, and she was subsequently referred to ED for further assessment.

Examination

**Figure 1:**
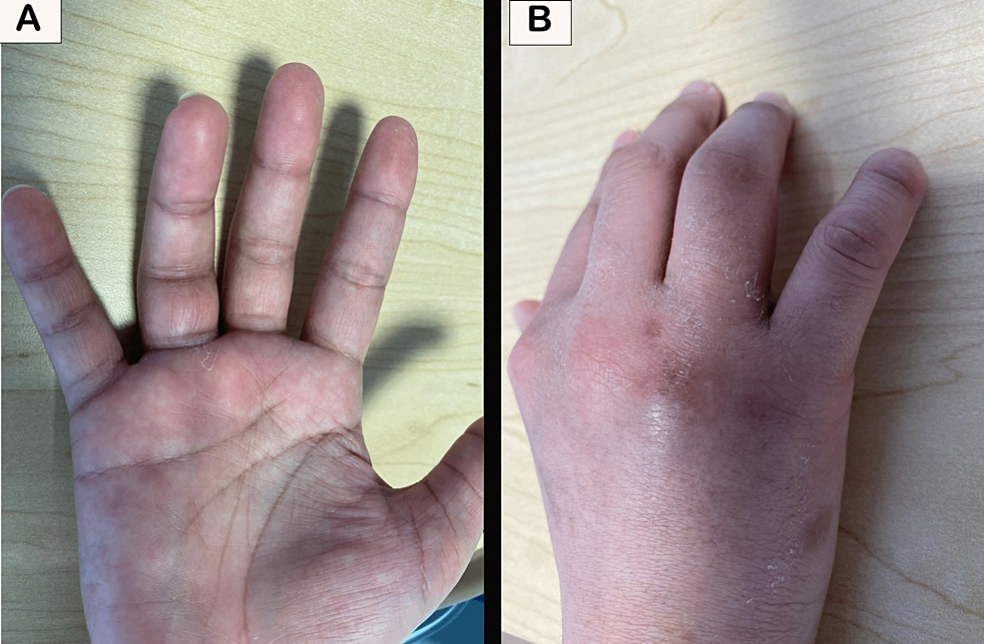
Patient’s right hand on clinical examination. The ring (4th) finger is markedly swollen and maintained in a flexed position. The old puncture wound from the ‘fight bite’ injury is visible at the dorsal base of the ring finger overlying the Metacarpophalangeal joint. MCP: Metacarpophalangeal

On arrival in ED, the patient was evaluated and found to have a temperature of 37°C and otherwise normal vital signs, with a chief complaint of worsening pain over the right-hand ring finger and associated swelling (figure 1). No other symptoms, such as fever, were reported. The pain was reproduced with finger extension, and the ring finger was swollen along its entire length, with mild redness, and held in a flexed position during examination. An old, healed wound over the dorsal aspect of the Metacarpophalangeal (MCP) joint of the right ring finger was noted with joint swelling, tenderness, and surrounding soft tissue edema noted on examination.

In addition to the findings above, tenderness was elicited over the right ring finger’s flexor aspect. The pain was reproduced on the passive extension of the finger, raising suspicion of underlying flexor tenosynovitis. On questioning, the patient initially reported that she fell down some stairs three weeks ago but later revealed that the injury was sustained from an altercation as described above.

Investigations

Initial laboratory investigations were generally unremarkable for an acute inflammatory state, with a White Blood Cell count of 7.59 × 109/L, C-reactive Protein (CRP) of 1.55 mg/L, and procalcitonin of 0.049 ng/ml.

**Figure 2:**
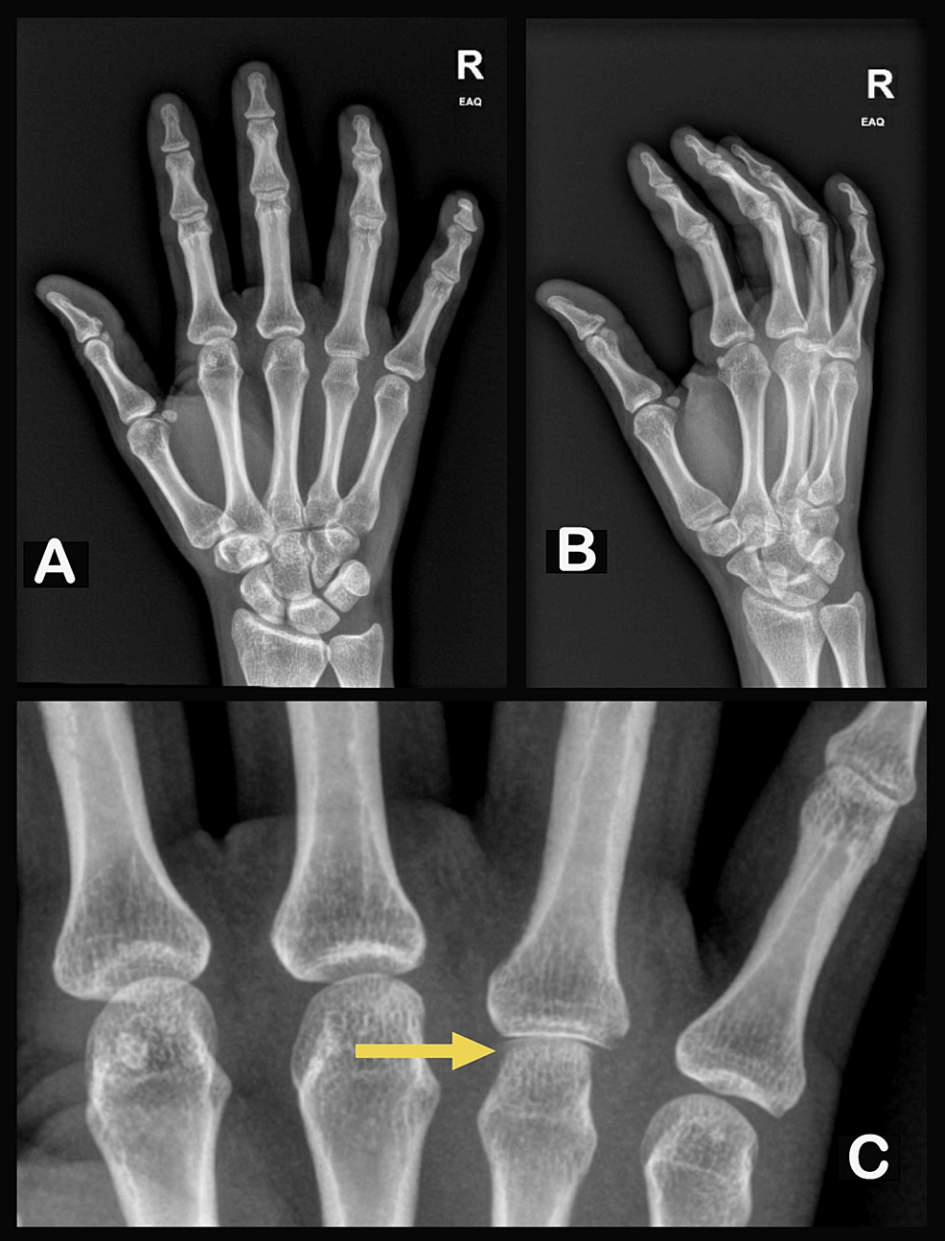
X-ray of the right hand. The bony effacement and periosteal elevation (C) at the MCP region of the fourth finger suggest osteomyelitis. MCP: Metacarpophalangeal

X-rays of the hand (figure 2) revealed changes consistent with osteomyelitis of the fourth metacarpal head, showcased by effacement of the lateral aspects of the bony head along with a focal point of periosteal elevation. The patient was admitted, and a subsequent ultrasound of the fourth finger and MCP joint demonstrated moderate flexor and extensor tenosynovitis of the ring finger, along with synovitis of the metacarpophalangeal joint and no drainable effusion.

Treatment

During admission under Plastic Surgery, the ring finger was splinted to maintain immobilization. The pain was controlled with intravenous (IV) acetaminophen every 6 hours as needed, and antibiotics (IV amoxicillin-clavulanate 1000mg every 8 hours) were administered for five days in the hospital. The finger pain and swelling gradually improved, and the external wound healed with no discharges. No operative intervention or joint washout was required for the patient. The finger splint was kept in place until discharge.

Outcome

The patient’s hand function and pain adequately responded to antibiotic therapy over the five days of admission. Seven additional days of oral amoxicillin-clavulanate were prescribed upon discharge, followed up in the Plastic Surgery clinic in one week, and safety net instructions to return to the ED in the event of worsening pain, fever, or increasing swelling. Unfortunately, she did not return for an ED visit or an outpatient appointment for further evaluation.

Verbal and written consent was obtained from the patient permitting the dissemination of information from their clinical encounter. A copy of the signed consent form is available upon request.

## Discussion

Epidemiology

Animal and human bite wounds constitute approximately 1% of all U.S. ED visits [2]. Although dogs and cats are implicated in most mammalian bites, human bites represent a significant subset of patients with a high risk of infection and disability from wounds if not appropriately treated [3].

Most human bite patients are young adults, with notable male predominance, and facial and upper limb regions are more commonly involved. Injuries tend to occur more frequently on weekends and public holidays, with preceding alcohol consumption reported in up to 86% of cases [4]. Many patients either provide unreliable histories due to intoxication or underreport the presence or extent of injury due to fear of embarrassment or legal consequence [1].

Pathophysiology

Human bites are of two types based on the mechanism of injury - occlusion bites or clenched/closed fist bites (commonly referred to as “fight bites”) [5]. Occlusion bites describe direct, often intentional ‘biting down into the victim’s skin, usually the finger or hand, with a strong enough force to produce a break in the skin. “Fight bites” occur when a closed fist impacts the teeth in another person’s mouth, often because of an unintentionally placed punch. This results in an injury over the dorsal aspect of the MCP joints, typically including the third (middle) finger joint. More than 50% of injuries penetrate the MCP joint, extending into the extensor tendon, cartilage, and even bone [5].

Infections occur in around 10-15% of human bites and are often polymicrobial. Culprit microorganisms often inoculate from oral flora to a clenched fist during the bite. Penetration typically involves the extensor mechanism and joint capsule underlying the dorsal aspect of the MCP joint in a clenched fist, with relatively little soft tissue involvement. Furthermore, in the anatomical position when the fingers are extended, the injured extensor tendon is not visible through the skin wound, which may seal the articular surface, encouraging bacterial growth and spreading to soft tissues (cellulitis, abscesses), joint (septic arthritis), bone (osteomyelitis) and palmar and flexor tendon sheaths (flexor and extensor tenosynovitis) [6].

Tendons, ligaments, bone, and cartilage are relatively avascular, predisposing them to a higher risk of infection. Commonly isolated species of bacteria include aerobic (e.g., Streptococcus, Staphylococcus, Eikenella corrodens, Haemophilus) and anaerobic (Prevotella, Fusobacterium, Eubacterium) species [6]. Candida species are more frequently encountered in occlusion bites. HIV, Hepatitis B (HBV), and Hepatitis C (HCV) are blood-borne illnesses that may transfer across individuals due to fight bites when significant blood is exposed [7]. Each party’s HIV and hepatitis infection status should be explored, and appropriate post-exposure prophylaxis (PEP) should be commenced. In our case, while a source panel was requested, it was not confirmed whether it was performed as the person was incarcerated under police custody at the time. The patient, however, underwent testing and was negative for HIV and hepatitis serologies.

Human bites pose a negligible risk of HIV transmission; hence empiric HIV PEP is not routinely indicated [8]. However, it should be considered where the assailant is known to be HIV-positive and the injury has involved possible blood transfer. Where doubt exists, immediate consultation with the local Infectious Disease department is recommended.

Delays in a presentation to healthcare facilities or failure to initiate appropriate management, including antibiotic coverage, are associated with a higher risk of severe infection [9]. Additionally, joint involvement, penetration below the dermis level, immunocompromised status, and injuries with concomitant fractures require antibiotic coverage due to higher infection rates [9].

Diagnosis

Fight bite injuries may present as seemingly benign puncture marks on examination but are frequently associated with extensive infection if missed, warranting an investigative history and physical examination with a high index of suspicion for occult injury [10].

Our patient presented with signs suggestive of flexor sheath infection. Allen B. Kanavel first described the components of this sign in 1921, eponymously described after that as Kanavel’s signs [11], where flexor tenosynovitis is confirmed clinically through the presence of 1) tenderness over the flexor sheath of the affected finger, 2) flexed position of the finger, 3) pain on passive extension of the finger, more at proximal aspect, and 4) symmetrical, fusiform swelling involving the entire finger [11].

Standard plain film radiography is utilized to evaluate underlying bony involvement, particularly at the proximal phalanx and MCP joint. Ultrasonography may evaluate fluid collection within the joint and underlying vascular insults.

Treatment

ED management of a fight bite injury consists of wound care, bleeding and pain control, cleansing and irrigating the wound, and removing debris through washout. It is essential to commence IV antibiotics early, and surgical consultation is indicated in cases when penetration is suspected to be significantly below the dermis.

Wounds are seldom closed early due to a high risk of development of infection [12]. Regular follow-up or inpatient examination is warranted. Antibiotic prophylaxis, typically with amoxicillin-clavulanate, is prescribed to cover a broad spectrum of causative bacteria. Tetanus prophylaxis and Hepatitis B vaccine in unvaccinated individuals are also advised. Operative intervention is often avoided if appropriate antibiotics and aggressive irrigation are commenced early, within 24 hours of injury [12].

Complications

The MCP joint is a synovial, condyloid joint with proximity to structures responsible for finger movement, including flexor and extensor sheaths and collateral ligaments [13]. Without timely intervention, fighting bite-related septic arthritis can cause bone and soft tissue degradation within the MCP joint, resulting in significant loss of function, disability, and even amputation [5]. Osteomyelitis and necrotizing fasciitis may occur, traditionally associated with Staphylococcus and Streptococcus species [5,14].

Our patient developed progressive infection with deterioration of extensor and flexor tendon sheaths along with bone, as evidenced by features of osteomyelitis on X-ray (see Figure 2). Periosteal elevation, loss of articular surface, and smoothened cortex of bone are common radiographic features of bony degradation from osteomyelitis [15].

## Conclusions

Our case highlighted a cautionary tale of the consequences of delayed diagnosis and delayed or inappropriate antibiotic coverage for human fight bite injuries. Fight bite wounds may often appear benign on initial examination. However, a high index of suspicion for infiltration of deep, underlying structures is needed during the evaluation and consideration of aggressive wound irrigation and appropriate antibiotic coverage for suspected patients. These cases should be considered for urgent referral to a hand surgeon. Early antibiotic initiation within 24 hours on the first visit would have effectively impeded this patient’s progression to severe disease. Furthermore, this case highlights the clinical and radiographic signs of infection in ligament and bone from septic arthritis of the MCP joint.
